# Identification of an Autophagy-Related Risk Signature Correlates With Immunophenotype and Predicts Immune Checkpoint Blockade Efficacy of Neuroblastoma

**DOI:** 10.3389/fcell.2021.731380

**Published:** 2021-10-21

**Authors:** Wenjuan Kang, Jiajian Hu, Qiang Zhao, Fengju Song

**Affiliations:** ^1^Department of Epidemiology and Biostatistics, Key Laboratory of Molecular Cancer Epidemiology, Tianjin, National Clinical Research Center of Cancer, Tianjin Medical University Cancer Institute and Hospital, Tianjin, China; ^2^Tianjin Key Laboratory of Cancer Prevention and Therapy, Department of Pediatric Oncology, National Clinical Research Center for Cancer, Tianjin’s Clinical Research Center for Cancer, Tianjin Medical University Cancer Institute and Hospital, Tianjin, China

**Keywords:** neuroblastoma, autophagy, survival, gene, immune

## Abstract

Neuroblastoma is one of the malignant solid tumors with the highest mortality in childhood. Targeted immunotherapy still cannot achieve satisfactory results due to heterogeneity and tolerance. Exploring markers related to prognosis and evaluating the immune microenvironment remain the major obstacles. Herein, we constructed an autophagy-related gene (ATG) risk model by multivariate Cox regression and least absolute shrinkage and selection operator regression, and identified four prognostic ATGs (BIRC5, GRID2, HK2, and RNASEL) in the training cohort, then verified the signature in the internal and external validation cohorts. BIRC5 and HK2 showed higher expression in MYCN amplified cell lines and tumor tissues consistently, whereas GRID2 and RNASEL showed the opposite trends. The correlation between the signature and clinicopathological parameters was further analyzed and showing consistency. A prognostic nomogram using risk score, International Neuroblastoma Staging System stage, age, and MYCN status was built subsequently, and the area under curves, net reclassification improvement, and integrated discrimination improvement showed more satisfactory prognostic predicting performance. The ATG prognostic signature itself can significantly divide patients with neuroblastoma into high- and low-risk groups; differentially expressed genes between the two groups were enriched in autophagy-related behaviors and immune cell reactions in gene set enrichment analysis (false discovery rate *q* -value < 0.05). Furthermore, we evaluated the relationship of the signature risk score with immune cell infiltration and the cancer-immunity cycle. The low-risk group was characterized by more abundant expression of chemokines and higher immune checkpoints (PDL1, PD1, CTLA-4, and IDO1). The risk score was significantly correlated with the proportions of CD8+ T cells, CD4+ memory resting T cells, follicular helper T cells, memory B cells, plasma cells, and M2 macrophages in tumor tissues. In conclusion, we developed and validated an autophagy-related signature that can accurately predict the prognosis, which might be meaningful to understand the immune microenvironment and guide immune checkpoint blockade.

## Introduction

Neuroblastoma (NB) is one of the malignant solid tumors with the highest mortality in childhood. With malignant cells originating from immature nerve tissue in the adrenal glands, neck, chest, or spinal cord, this disease shows great heterogeneity in clinical and biological behavior (Elizabeth et al., 2014). Most NBs metastasize and then have a poor prognosis, whereas, in some low-age patients, the malignancy tends to show spontaneous regression. The prognosis is significantly correlated with multiple factors, including the age of diagnosis, site of onset, International Neuroblastoma Staging System (INSS) stage, MYCN amplification status, and DNA ploidy. Furthermore, Ikeda et al. (2002) pointed out that INSS stage 4 was the most significant poor prognostic factor after comparing the hazard ratio with other factors. The long-term survival of patients in INSS stage 4 remains around 50% though receiving chemotherapy, radiotherapy, and radical surgery (Matthay et al., 2016). Therefore, it is a subject worthy of research on the differential genes between INSS stage 4 and other stages.

Moreover, unlike most adult cancers, the total somatic mutation rate of NB is less than 20% (Andersson et al., 2020). Specific drugs targeting NB’s mutations are still being developed (Schulte et al., 2013; Duong et al., 2017; Lucas et al., 2020). On the other hand, aiming to awaken antitumor immunity, cancer immunotherapy is gradually becoming a leading issue across various cancer types, including NB treatment (Park and Cheung, 2020). However, immunotherapy is still in the early stages of development for pediatric solid malignancy, though widely recognized and applied in a variety of adult cancers nowadays (Lorenzo et al., 2018; Judith et al., 2021). Anti-PD-L1 and anti-CTLA-4 are now available in molecular studies and clinical treatment of NB (Priya et al., 2018). However, different from the adult tumors easily benefiting from immunotherapy, NB was reported to be resistant to immunotherapy due to its low immunogenicity, insufficient MHC-I expression, and few tumor-infiltrating lymphocytes in the microenvironment. It has been an attractive strategy to convert the immunosuppressive environment in the “cold tumor” into an immunostimulated one, mainly called a “hot tumor” (Judith et al., 2021). Clearly, the current clinical or biological factors are limited to judge the reaction in NB’s microenvironment; we need more effective biomarkers to guide immune reaction, including immune checkpoint blockade therapy (Whittle et al., 2017).

Macroautophagy (hereafter referred to as autophagy) is a ubiquitous self-degradation process during starvation or stress (Kondo et al., 2005). More and more researches have revealed the important relationship between autophagy and immunity of malignancy (Shibutani et al., 2015). As a double-edged sword, autophagy could promote immune evasion of pancreatic cancer by degrading MHC-I, whereas it may play a pivotal role in facilitation in the antigen presentation (Zeng et al., 2019; Yamamoto et al., 2020; Ynebrten, 2020). Moreover, there is increasing evidence of the role of autophagy-related genes (ATGs) in the development of NB. For example, high expression of beclin 1 is associated with a poor prognosis for NB (Belounis et al., 2016). Meanwhile, ATGs and long noncoding RNAs are differentially expressed between INSS stage 4 and INSS stage 4s NB, which highly indicates that autophagy plays an important role in spontaneous regression (Xinyao et al., 2020). Therefore, the net outcome between autophagy and immune in NB needs to be evaluated, as well as identifying the key prognostic ATGs that regulate the microenvironment. In the present study, we firstly compared the ATGs expression between INSS stage 4 and other stages of NB patients from Gene Expression Omnibus (GEO) datasets, identified four ATGs significantly associated with the prognosis, and subsequently constructed an ATG risk signature that revealed the close relationship with clinical prognostic factors. Finally, we discovered the significant association between ATGs risk signature and NB immune microenvironment to guide the clinical appliance of precise immunotherapy.

## Materials and Methods

### Clinical Samples and Data Acquisition

Gene expression profiles and clinical data were downloaded from GEO (Ron et al., 2002) (GEO^1^) GSE62564 (Su et al., 2014) (GPL11154) and ArrayExpress (Awais et al., 2018)^2^ database E-MTAB-8248 (Agilent-020382 Human Custom Microarray 44k). The survival data were acquired from the R2 genomics analysis and visualization platform^3^. Specifically, a total of 498 NB patients, who were in GSE62564 containing both RNA-seq and clinical data (INSS stage, age, MYCN amplification status, and progression), were randomly assigned into a training cohort (350 patients: 220 INSS stage No-4, 130 INSS stage 4) and an internal validation cohort (148 patients: 95 INSS stage No-4, 53 INSS stage 4) at a 7:3 ratio by using the “caret” package (Kuhn, 2015), version 4.0.3^4^ in R language. Two hundred twenty-three patients in E-MTAB-8248 possessing both gene expression profile and clinical data (INSS stage, age, MYCN status, and progression) composed the external validation cohort (134 INSS stage No-4, 89 INSS stage 4) (Table 1). Expression profiles, messenger RNA (mRNA) expressions for a total of 721 NB samples, were presented by log2 conversions. Meanwhile, we deleted genes whose expression was zero in all samples.

**TABLE 1:** Clinicopathological characteristics of samples in cohorts.

Clinicopathological characteristics	Number of samples		
	Training cohort (*n* = 350)	Internal validation cohort (*n* = 148)	External validation cohort (*n* = 223)
**INSS stage**			
No-4	220	95	134
4	130	53	89
**Age**			
≥18 months	140	58	120
<18 months	210	90	103
**MYCN status**			
Not Amplified	287	114	176
Amplified	58	34	46
NA	5	0	1
**Progression**			
Yes	136	47	89
No	214	101	134

### Tumor Cell Line Data Acquisition

Microarray data of NB cell lines were obtained from the GEO database under accession number GSE19274 (Cole et al., 2011) including 29 cell lines, 38 samples in total. After deleting the samples without data of MYCN status, there were 15 MYCN amplified and 10 MYCN nonamplified NB cell lines left.

### Identification of Autophagy-Related Messenger RNAs in Neuroblastoma

A total of 694 autophagy-related encoding genes were obtained from the Human Autophagy Database (HADb^5^) and the Molecular Signatures Database of Gene Set Enrichment Analysis (GSEA^6^). Next, 643 ATG mRNAs were identified by mRNAs of the training cohort intersected with the autophagy-related encoding genes list. Principal component analysis (PCA) was used to figure out the distribution between INSS stage No-4 and INSS stage 4 by using the ‘‘factoextra’’^7^ and ‘‘FactoMineR’’ package^8^. Then, differentially expressed mRNAs (DEmRNAs) were identified using the “limma” package, which criteria were mRNAs with | log2(fold-change)| ≤1 and false discovery rate < 0.05.

### Construction and Evaluation of an Autophagy-Related Gene Prognostic Signature

DEmRNAs were subject to the least absolute shrinkage and selection operator (LASSO) regression analysis for further screening out. Multivariate Cox regression analysis was subsequently applied for reserved genes from LASSO regression analysis to select candidate DEmRNAs for the risk signature. The risk score was calculated using the following formula:


$$Riskscore=\sum_{i=1}^{n}EXPi\times Coei$$

where EXP and Coe meant that the expression value and the regression coefficient of DEmRNAs from multivariate Cox regression analysis, respectively. Then, patients with their corresponding calculated risk score were divided into low- and high-risk prognostic groups according to the median value. Both LASSO and multivariate Cox regression analysis were conducted using the “survival” and “glmnet” packages.

Subsequent Kaplan–Meier (K-M) survival curves (“survminer” package) and the receiver operating characteristic (ROC) curve (“timeROC” package) were generated to determine the prognostic and predictive efficacy of DEmRNA-based risk signature.

### Internal and External Verification of Signature

To verify the ATG signatures, we calculated the risk score of each patient in the internal validation cohort using the method mentioned earlier. The log-rank test was then used to assess the overall survival (OS) difference between the two groups and plot the K-M survival curve. Finally, the ROC curve was drawn, and the area under the curve (AUC) of predicting prognosis was calculated. External validation was done with the same method discussed earlier by extracting ATG DEmRNAs from the training cohort.

### Evaluation of the Relationship Between Signature and Clinical Characteristics

We evaluated the relationship between ATG signature and clinical characteristics. By dividing the NB samples into a high-risk group and a low-risk group based on the ATG signature’s risk score. We further analyzed the relationship between risk score and clinical variables, including fustat (alive, dead), age (≥18 months, < 18 months), INSS stage (4, No-4), MYCN status (not amplified, amplified), and progression (yes, no). Finally, the relationship was further evaluated in the internal and external validation cohort.

### Construction and Evaluation of Nomograms

In the training cohort, we combined the clinical characteristics with ATG signature to use univariate and multivariate Cox proportional hazards regression model to assess the association between the variables and OS of NB patients. Then, we selected the independent risk factors to construct a nomogram (“rms” package) for easier clinical use. We drew calibration charts to evaluate the accuracy of the nomogram. Also, the C-index, the ROC curves, integrated discrimination improvement (IDI), and net reclassification improvement (NRI) (“survIDINRI” package) were used to assess the performances of the nomogram.

### Functional Enrichment Analysis

Gene Set Enrichment Analysis (GSEA)^9^ was used to interpret gene expression data in the biological functions and pathways (Subramanian et al., 2005). This method derives its function by analyzing gene sets, so it can be used to determine whether the gene set shows a statistically significant difference between the two risk states. In all enrichment analyses, the adjusted *p*-values < 0.05 were considered as significant results.

### Evaluation of Immune Cell Infiltration and Cancer-Immunity Cycle

We used the Estimation of Stromal and Immune cells using Expression data (“estimate” package) algorithm to calculate immune and stromal scores to clarify the difference between the high-low risk groups and further determine the relationship between the ATG-prognostic model and the immune microenvironment.

Subsequently, CIBERSORT was used to infer the relative proportion of 22 infiltrating immune cells in each sample of the two risk groups (Newman et al., 2015). The algorithm of 1,000 permutations was adopted. Only samples with a *p-*value < 0.05 in CIBERSORT analysis were included to perform the subsequent analysis of comparing differential immune infiltration levels between the subgroups grouped by risk scores. Also, we clarified the order of the components of immune cells (“ggplot2” package), correlation analysis (“PerformanceAnalytics” packages).

For immune activity profiling, we applied the Tumor ImmunoPhenotype (TIP)^10^ to estimate the activity level, especially the Step4 (trafficking of immune cells to tumors) (Liwen et al., 2018). The expression of classical immune checkpoint genes as immunotherapeutic targets including CTLA4, IDO1, PD-1, and PD-L1 and the expression of human leukocyte antigen (HLA)-A, HLA-B, and HLA-C were investigated, respectively, in high-low risk groups.

### Statistical Analysis

Except for GSEA, all statistical analyses involved in the research were conducted through the R programming language (version 4.0.3). *p* < 0.05 was considered statistically significant.

## Results

### Construction and Definition of the Autophagy-Related Gene Signature

**FIGURE 1:**
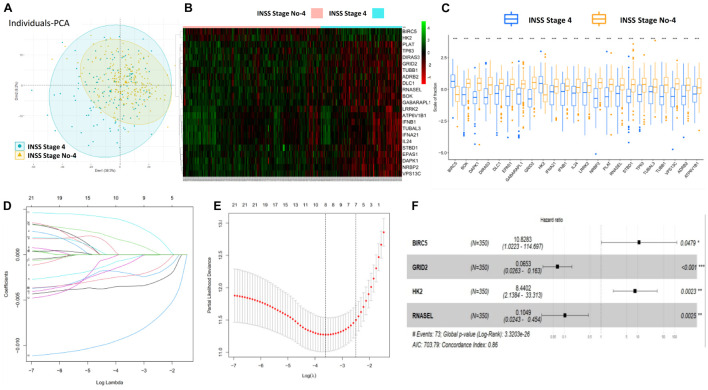
Construction and definition of ATG signature. **(A)** PCA of ATG mRNAs between INSS stage No-4 and 4. **(B,C)** Twenty-three DEmRNAs were shown with median expression levels. **(D,E)** Optimized lambda determined in LASSO regression model, with min lambda 0.0270, there were eight indexes selected. **(F)** Multivariate Cox regression analysis confirmed four potential prognostic ATG DEmRNAs as independent factors.

Firstly, the flowchart briefly introduced the process and design of the study (Supplementary Figure 1). Totally, 643 ATG mRNAs were included in this study. PCA was used to figure out the distribution of these ATG mRNAs between INSS stage No-4 and INSS stage 4 graphically (Figure 1A). To reflect the distinct features of INSS stage No-4 and INSS stage 4, 23 DEmRNAs between these two groups were selected for further study and shown with median expression levels (Figures 1B,C).

After the LASSO regression analysis, with the min lambda 0.0270, eight indexes were included in subsequent multivariate Cox regression analysis (Figures 1D,E). As a result, four potential prognostic ATG DEmRNAs were screened out. BIRC5 and HK2 were regarded as risk factors (HR > 1), whereas GRID2 and RNASEL were found to be protective (HR < 1) (Figure 1F).

**FIGURE 2:**
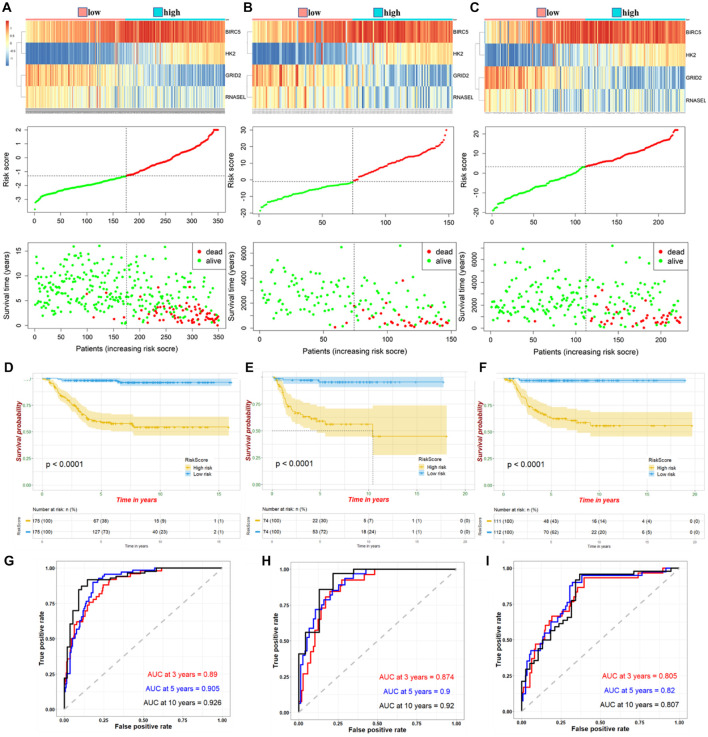
Expression of high- and low-risk groups under ATG prognostic risk signature in samples. Expression heatmap of identified four ATGs and corresponding risk score and survival status of patients in training cohort **(A)**, internal **(B),** and external validation **(C)**. Kaplan–Meier curve for high- and low-risk group patients in training cohort **(D)**, internal **(E),** and external validation **(F)**. Time-dependent ROC curve of risk score in training cohort **(G)**, internal **(H),** and external validation **(I)**.

The four ATG DEmRNAs were integrated to establish a risk signature in the training cohort. Samples in the training cohort were divided into high- and low-risk groups according to the median risk score. As shown in Figure 2A, the BIRC5 and HK2 expression levels were positively related to the risk score, whereas the GRID2 and RNASEL expression levels were opposite. Meanwhile, an increased risk score suggested more deaths. The K-M curve indicated a significantly poorer prognosis in the high-risk group than that in the low-risk group (Figure 2D). Besides, the AUCs for 3−, 5−, and 10-year survival were 0.89, 0.905, and 0.926, respectively, suggesting good predictive performances of this ATG signature (Figure 2G).

### Verification of Autophagy-Related Gene Prognostic Risk Signature With Internal and External Cohorts

According to the median risk score, NB samples were divided into high- and low-risk groups to perform tests in internal and external validation cohorts. Consistent with the results in the training cohort, upregulation of BIRC5 and HK2, downregulation of GRID2 and RNASEL expression, and correspondingly increased number of deaths were observed with increased risk score (Figures 2B,C). The K-M curves showed a relatively more favorable prognosis in the low-risk group than in the high-risk group (Figures 2E,F). AUCs for the 3−, 5−, and 10-year survival were 0.874, 0.9, and 0.92, respectively, of the internal validation. Meanwhile, AUCs for the 3−, 5−, and 10-year survival were 0.805, 0.82, and 0.807, respectively, of the external validation (Figures 2H,I). These further suggested a good predictive efficacy of the ATG risk signature.

### Hub Autophagy-Related Genes of the Signature Associate With MYCN Status

**FIGURE 3:**
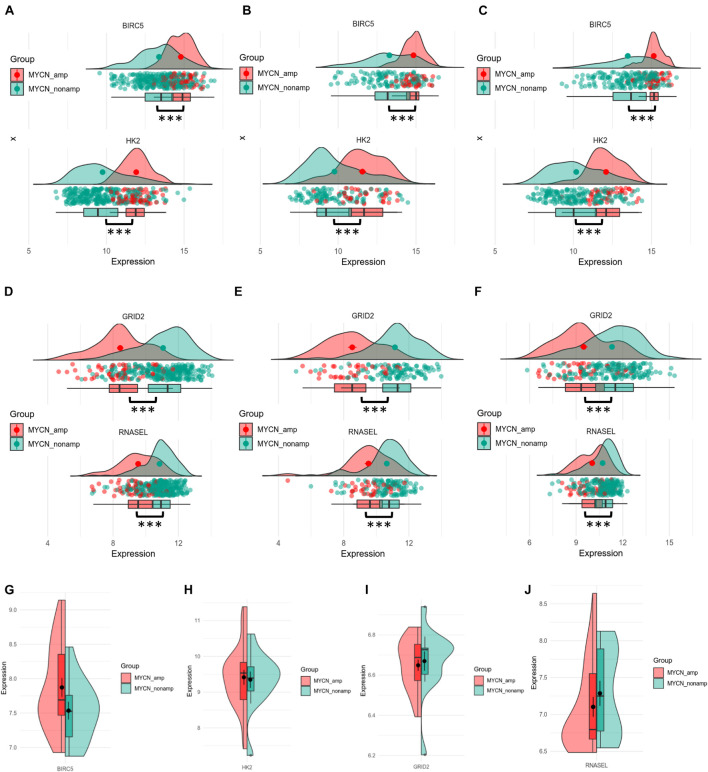
Differential expression of four hub ATGs between MYCN amplification and MYCN nonamplification in tissue samples and cell lines. Different expression of BRIC5 and HK2 in tissue samples between MYCN amplification and MYCN nonamplification of training cohort **(A)**, internal **(B)**, and external validation **(C)**. Different expression of GRID2 and RNASEL in tissue samples between MYCN amplification and MYCN nonamplification of training cohort **(D)**, internal **(E)**, and external validation **(F)**. Different expression of BRIC5, HK2, GRID2, and RNASEL between MYCN amplification and MYCN nonamplification in NB cell lines **(G–J)**.

MYCN proto-oncogene amplification, an important prognostic factor for NB, always predicts highly malignant disease and poor outcomes. To explore the potential relationship between up- or downregulated ATGs with MYCN status, the different gene expression of each ATG of the signature between MYCN amplified and MYCN nonamplified groups was computed. As shown in Figures 3A–C, BIRC5 and HK2 had significantly higher expression levels in MYCN amplified group compared with MYCN nonamplified group in training (345 patients: 58 MYCN amplified, 287 MYCN nonamplified), internal (148 patients: 34 MYCN amplified, 114 MYCN nonamplified), and external cohorts (222 patients: 46 MYCN amplified, 176 MYCN nonamplified) (*p* < 0.001). Meanwhile, GRID2 and RNASEL showed the opposite trends (*p* < 0.001) (Figures 3D–F). Further comparison analysis in the NB cell lines datasets showed that BIRC5 and HK2 were significantly enriched in the MYCN amplified cell lines, whereas GRID2 and RNASEL were enriched in the MYCN nonamplified cell lines (Figures 3G–J). These outcomes also demonstrated that BIRC5 and HK2 were regarded as risk factors, whereas GRID2 and RNASEL were deemed to be protective.

### Correlation Between Autophagy-Related Gene Signature and Clinical Characteristics and Then Construction of Nomograms

**FIGURE 4:**
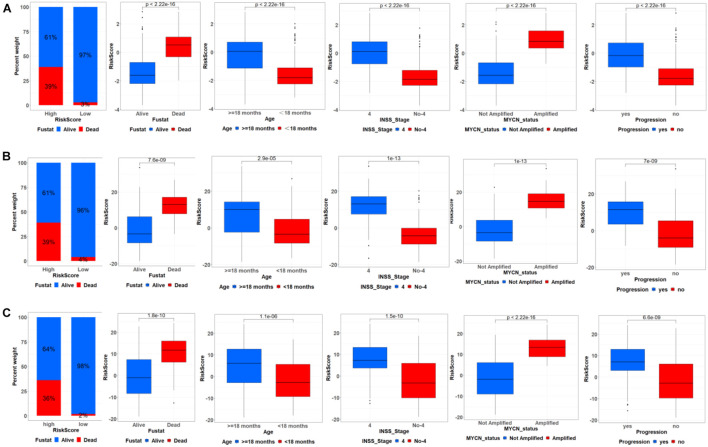
Correlation between ATG signature and clinical characteristics. Relationship between ATG signature and clinical characteristics including outcome, age, INSS stage, MYCN status, and progression in training cohort **(A)**, internal **(B)**, and external validation **(C)**.

We further analyzed the relationship between ATG signature and clinical characteristics. The samples were divided into high- and low-risk groups based on the median risk score. As shown in Figure 4A, in the training cohort, ATG risk signature can predict the OS of patients in different subgroups. The patients with the higher risk score had the lower OS. Respectively, patients with < 18 months, INSS stage No-4, or MYCN nonamplified had lower risk scores. In addition, patients in the metastasis group had higher ATGs risk scores. Meanwhile, the internal and external validation simultaneously recorded a similar correlation between ATG signature and clinical characteristics mentioned earlier (Figures 4B,C).

**FIGURE 5:**
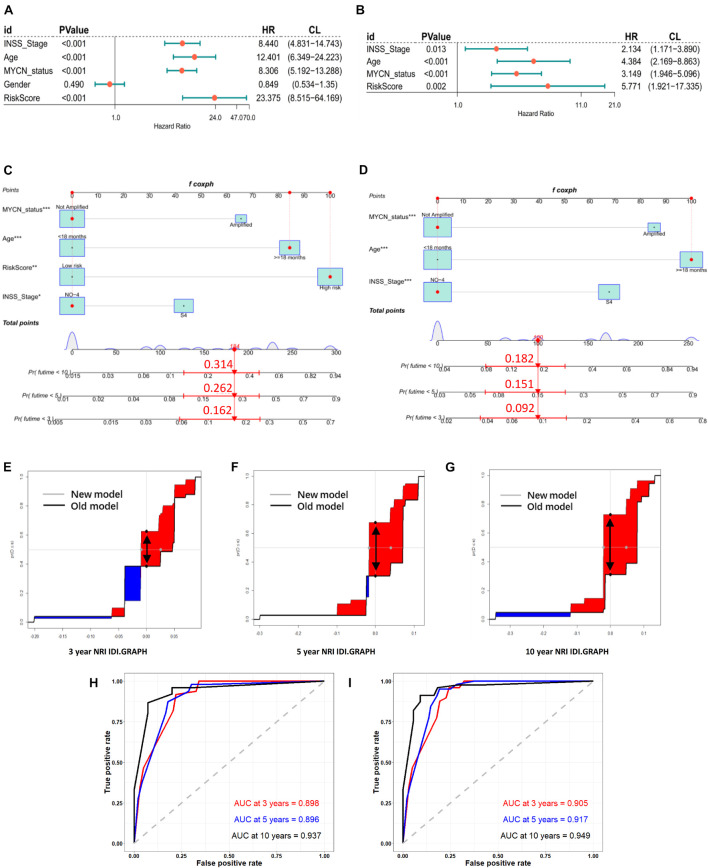
Models of risk score and clinical variables were established, compared, and verified. Forest plot of univariate **(A)** and multivariate **(B)** Cox regression results of RiskScore (define ATGs risk signature as a variable) and clinical variables. Establishment of nomograms including new models (RiskScore plus) **(C)** and old models (clinical variables only) **(D)**. NRI and IDI for 3- **(E)**, 5- **(F),** and 10- **(G)** year graph in new models (RiskScore plus) and old (clinical variables only). NRI is showed as distance between two black spots (double arrow); IDI is showed as area difference between red part and blue part. ROC curves of old **(H)** and new **(I)** models for predicting 3-, 5-, and 10-year survival.

In the training cohort, univariate and multivariate Cox regression analyses showed that RiskScore (define ATG risk signature as a variable) and clinical variables including INSS stage, age, and MYCN status were independent risk factors for NB patients (Figures 5A,B). Then, we constructed nomograms and evaluated the accuracy of the new model (clinical variables plus RiskScore) (Figure 5C) and the old model (clinical variables only, without RiskScore) (Figure 5D). The C-indices based on the new nomogram (0.874) were higher than those based on the old one (0.864). Then, we randomly chose a patient to verify the predictive ability of the models. The general condition of this patient numbered gsm1529083 was as follows: male, INSS stage III, MYCN-not-amplified, > 18 months (age), 1,120 days (OS), high RiskScore, and dead. In Figure 5C (clinical variables plus RiskScore), the predicted OS<5 years was approximately 0.262. In Figure 5D (clinical variables only, without RiskScore), the predicted OS<5 years was approximately 0.151. Eventually, the OS time of gsm1529083 was approximately 3.06 years. The predicted survival rate was more accurate using the new model with clinical variables and the RiskScore. Compared with the old one, analysis of accuracy showed that the new model’s NRI for the 3-, 5-, and 10-year follow-ups were 1.2% (*p* < 0.001), 4.6% (*p* = 0.010), and 5.2% (*p* < 0.001), respectively. Similarly, the IDI for 3, 5, and 10 years were 24.0% (*p* < 0.001), 37.4% (*p* = 0.040), and 41.7% (*p* = 0.050), respectively (Figures 5E–G). As shown in the ROC curves, the AUCs of the new models for predicting the 3-, 5-, and 10-year survival were 0.9, 0.917, and 0.949, respectively, larger than those of the old models (Figures 5H,I). These results indicate that the new nomogram had a greater potential for accurately predicting long-term outcomes compared with the old one.

### Predicting the Functions and Pathways of the Autophagy-Related Gene Prognostic Signature in Neuroblastoma by Gene Set Enrichment Analysis

**FIGURE 6:**
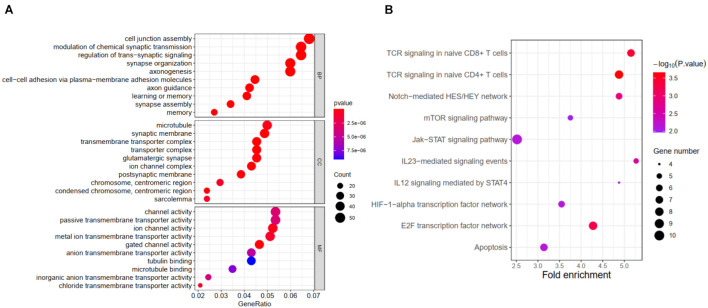
Results of GSEA for ATG signature. **(A)** Significantly enriched GO terms by GSEA. **(B)** Significantly enriched pathways by GSEA.

Further functional annotation was conducted through GSEA (Figures 6A,B). The results revealed that the differentially expressed genes between the two groups were enriched in autophagy-related and tumor-related behaviors, immune cell functions, and responses (false discovery rate q-value < 0.05). The results strongly indicated that the risk signature was closely associated with tumor immune microenvironment, a finding that deserved further analysis.

### Autophagy-Related Gene Prognostic Signature Reflecting Immune Cell Infiltration

**FIGURE 7:**
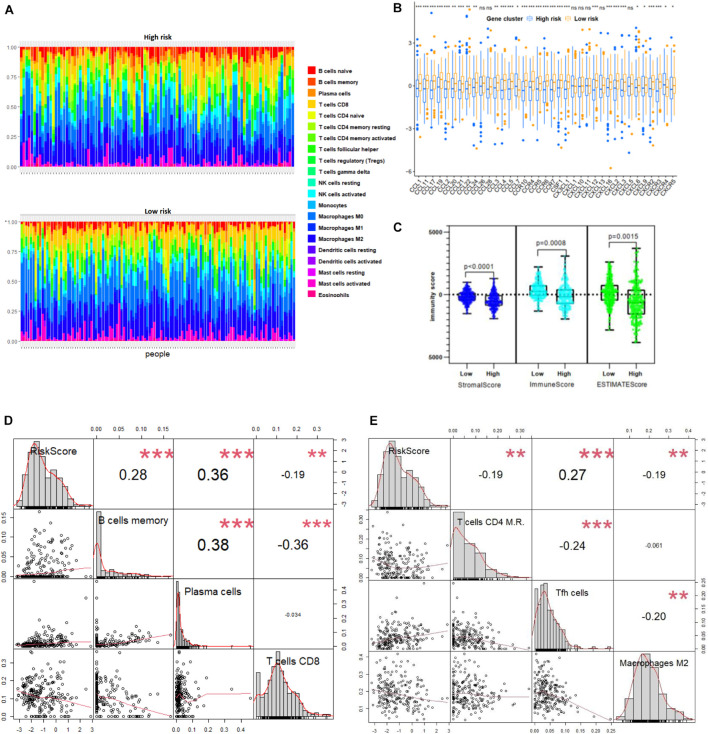
ATG signature reflecting immune cell infiltration. **(A)** Heatmap shows normalized absolute abundance of 22 immune cell types in individual samples of high- and low-risk group. **(B)** TIP to estimate activity scores of fourth step, trafficking of immune cells to tumors. **(C)** Degree of stromal, immune, and estimate score in high- and low-risk group. **(D,E)** Relationships between RiskScore and infiltration abundances of six immune cell types including B cells memory, plasma cells, T cells CD8, T cells CD4 memory resting (here T cells CD4 M.R.), Tfh cells, and macrophages M2 (*p* < 0.01).

We further correlated different immune risk groups and signature genes of various functional immune cell subtypes to explore the relationship between the ATG model and various infiltrating immune cell types. As mentioned earlier, using CIBERSORT in combination with the LM22 matrix, the distribution of immune cells of the high-risk and the low-risk NB samples divided by ATG signature was shown (Figure 7A). Furthermore, we used the Tracking TIP to estimate the activity scores of the fourth step, trafficking of immune cells to tumors, of the cancer-immunity cycle. Results from TIP showed low infiltration levels of the high ATG risk group compared with the low ATG risk group (Figure 7B). In addition, as shown in Figure 7C, the degree of the stromal score (*p* < 0.0001), immune score (*p* = 0.0008), and estimate score (*p* = 0.0015) were higher in the low-risk group than the high-risk group. Compared with patients in the high-risk group, the abundance of the cell populations in the patients in the low-risk group was higher in T cells CD8, T cells CD4 memory resting, and macrophages M2, whereas lower in T cells follicular helper, B cells memory, and plasma cells (Figures 7D,E). Overall, immune cell infiltration was negatively correlated with the ATG risk scores and indicated that patients from the low ATG risk group might have a higher immune activity and function on T cell regulation.

### Correlation Between Immune Checkpoint Genes and Risk Scores

**FIGURE 8:**
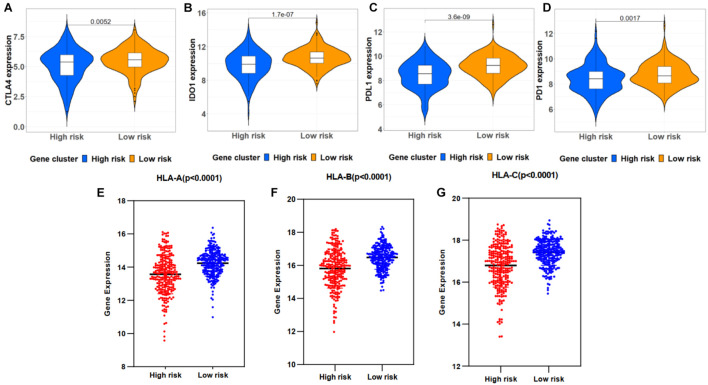
Correlation between immune checkpoint genes and risk scores. **(A–D)** Immune checkpoint gene expression, including CTLA4, IDO1, PD-L1, and PD-1, in high- and low-risk groups stratified by risk signature. **(E–G)** HLA-A, HLA-B, and HLA-C expression in two groups.

The correlations between the risk signature and immune checkpoint genes were investigated in high- and low-risk groups. As a result, patients in the low-risk group exhibited a relatively higher expression of CTLA4, IDO1, PD-1, and PD-L1 (Figures 8A–D). Moreover, HLA-A, HLA-B, and HLA-C showed a high proportion of the low-risk group (Figures 8E–G).

## Discussion

Considering NB as one of the most progressive solid cancer in children, it is urgently needed to identify reliable prognostic markers to guide the diagnosis and treatment. Autophagy has been considered a double-edged sword in molecular mechanisms of NB. The autophagy-related biological processes are highly enriched and supposed to act on the spontaneous regression of low-risk NB (Xinyao et al., 2020). Meanwhile, it mainly promotes cell proliferation and acts on the process of tumor resistance to chemotherapy (Wang et al., 2014; Yu et al., 2018). In various adult cancers, ATG-based signature has been constructed to predict the prognosis, not yet in NB. As the overall outcome for stages 1, 2, and 3 NB is now approximately 90–100%; however, the prognosis of NB patients with stage 4 is significantly worse than other stages, so the differential ATGs between stage 4 and no-stage 4 need to be explored (Ikeda et al., 2002; Agarwala et al., 2014). Thereby in this work, we focused on four hub ATGs after LASSO and multivariate Cox regression.

After screening for prognosis, BIRC5, GRID2, HK2, and RNASEL were identified as the hub component genes of ATG-based signature, which also could be considered as the therapeutic targets in the autophagy pathway. From the perspectives of tumor tissue and cell lines, we simultaneously found that BIRC5 and HK2 may increase tumor malignancy with MYCN amplification, whereas GRID2 and RNASEL were antagonistic to MYCN amplification. BIRC5 was reported to be induced *via* functional cooperation between MYCN and E2F1 (Isabella et al., 2009). Besides, MYCN might increase the expression of HK2 *via* the HNF4A-AS1 axis and finally promoted the tumorigenesis and aggressiveness of NB cells (Huajie et al., 2020). These consistent findings may provide innovation for revealing the transcription factor function and upstream and downstream targets of MYCN. Correspondingly, high expression levels of BIRC5 and HK2 were associated with poor prognosis in NB patients, whereas GRID2 and RNASEL were the opposite. Inhibition of the upregulated oncogenes has been one of the main means of targeted therapy (Fengzhi et al., 2019). Meanwhile, exploring the single nucleotide polymorphisms of genes with a good prognosis is also helpful to judge the prognosis in childhood cancer (Seth et al., 2016).

Notably, BIRC5 has been explored as one of the hub ATGs in the prognostic signatures of breast cancer, prostate cancer, and human endometrial cancer, as well as GRID2 in gastric cancer and endometrial carcinoma (Yunyan et al., 2015; Hu et al., 2020; Qiu et al., 2020; Zhang et al., 2020; Chen et al., 2021). The reports discussed earlier suggested that these two hub ATGs may play an important role in solid pan-cancer. Besides, GRID2 is associated with a good prognosis and may be related to neural death and neurodegenerative changes (Fekrije et al., 2003; Zeynep et al., 2016). On the contrary, HK2 regulated autophagy and played a significant role in shaping the malignant phenotype of NB and promoting the progression of this disease (Botzer et al., 2016). RNASEL triggered autophagy in response to viral infections and was associated with DNA damage and apoptosis of cancer cells (Chakrabarti et al., 2012; Huijing et al., 2019). In this study, the combined application of the four hub ATGs in the immune microenvironment of NB was first discovered.

Based on the expression of four ATGs, we constructed a novel prognostic signature and then evaluated the predictive power of the model through internal and external validation. The prognosis of the high-risk group was significantly worse than that of the low-risk group. The expression trends of the four hub ATGs were similar in different validation cohorts. In different cohorts, the time-dependent ROC suggested that these four ATG signatures had considerable prognostic accuracy in predicting the 3-, 5-, and 10-year survival rates. Moreover, the high-risk score was significantly correlated with clinical poor survival predictors such as older age, advanced stage, and MYCN amplification and related to disease progression. As Figure 5 shows, we used multivariate Cox regression to screen variables and subsequently constructed a nomogram with or without risk score. Patient gsm1529083 was randomly selected from the GSE62564 database, the nomogram (Figure 5C), including the gene risk score, showed more accuracy and practicality. In addition to the commonly considered AUC method, we used NRI and IDR methods to evaluate the efficiency differences between nomograms, which also directly revealed that a new nomogram with the four hub ATG risk score was more advantageous (Maarten et al., 2014; Hayashi and Eguchi, 2019).

From the results of gene function enrichment, the differential genes between groups were closely related to immunity reactions. They were mainly enriched in T cell development, interleukin function, and metabolism, such as the mTOR pathway, which further encouraged us to combine the autophagy signature with immunity. We found that the low-risk group had widely elevated chemokines, which may reverse the “cold tumor” in the immunosuppressive state to be a “hot tumor.” Lieke et al. reported that immune cells expressed a variety of chemokine receptors for chemotaxis and selectin ligands to bind to blood vessels and migrate into surrounding tissues (Van et al., 2017). Therefore, it made logical sense that the low-risk group had a higher immune score than the high-risk group. We conducted a correlation test between the risk score and the composition of immune cells. Generally, as the main force in antitumor immunity of NB, T lymphocytes significantly inhibited tumor development and improve prognosis as expected (Mina et al., 2016). Proportions of CD8 T lymphocytes and CD4 memory resting lymphocytes were negatively correlated with the ATG risk score. Besides, B lymphocytes and T follicular helper cells also produce a marked effect in the microenvironment. Wenjun et al. reported that the subtype γδTFH cells were positively correlated with serum total immunoglobulin G and plasma cells in NB patients, and all the components mentioned earlier were significantly higher than in normal children (Mou et al., 2017). Last but not least, M2 macrophage was reported to be regulated by the mTOR pathway, an important autophagy-related pathway (Chen et al., 2020). “Re-educating” tumor-associated macrophages has been considered as a novel immunotherapy strategy for NB, whose potential could be evaluated by the present signature (Liu and Joshi, 2020). The immune checkpoints including PDL1, PD1, CTLA-4, and IDO1 were also analyzed. Interestingly, the expression of immune checkpoints was higher in the low-risk group, similar to several reports conducted by Jinhui et al. (2020) and Yaqiong et al. (2020). Meanwhile, HLA-related molecules were significantly upregulated in the low-risk group. Accompanied with PDL1, HLA was discovered to represent a novel prognostic biomarker for NB (Melaiu et al., 2017). The expressions of immune checkpoint and HLA complex were an important factor in determining the response to immune treatment such as anti-GD2 therapy (Navid et al., 2009; Sameer and Shakeel, 2017; Palanisamy et al., 2018).

There are still limitations in this study. Firstly, the clinical information downloaded from online databases is limited and incomplete, and biological factors such as chromosomal variation and individual therapeutic regimens are unavailable. Secondly, the biological functions of autophagy-related hub genes need to be examined by relevant function assay *in vitro* or *in vivo*. Thirdly, although the ATG signature model shows a powerful predictive function in prognosis, the effectiveness remains to be further verified in a multicenter, large-scale prospective study receiving immune checkpoint blockade.

## Conclusion

We have linked autophagy with the immune microenvironment in NB by screening and constructing a four-ATG signature, which, combined with clinical factors, can accurately predict the long-term outcomes. Moreover, the risk score generated by this model can be used as an indicator of immune cell infiltration and a guide for immune checkpoint blockade.

## Data Availability Statement

The datasets presented in this study can be found in online repositories. The names of the repository/repositories and accession number(s) can be found below: https://www.ncbi.nlm.nih.gov/geo/, GSE62564; https://www.ebi.ac.uk/arrayexpress/, E-MTAB-8248; https://hgserver1.amc.nl/cgi-bin/r2/main.cgi, R2 genomics; http://autophagey.Iu/clusterring/index.html, Human Autophagy Database; http://timer.cistrome.org, TIMER; http://biocc.hrbmu.edu.cn/TIP/index.jsp, Tumor Immuno Phenotype; and https://www.ncbi.nlm.nih.gov/geo/, GSE19274.

## Author Contributions

QZ and FS conceived and designed the experiments. JH obtained and assembled data. WK analyzed and interpreted the data. JH and WK wrote the manuscript. All authors read and approved the final manuscript.

## Conflict of Interest

The authors declare that the research was conducted in the absence of any commercial or financial relationships that could be construed as a potential conflict of interest.

## Publisher’s Note

All claims expressed in this article are solely those of the authors and do not necessarily represent those of their affiliated organizations, or those of the publisher, the editors and the reviewers. Any product that may be evaluated in this article, or claim that may be made by its manufacturer, is not guaranteed or endorsed by the publisher.

## Footnotes

1
http://ncbi.nlm.nih.gov/geo/

2
https://www.ebi.ac.uk/arrayexpress/

3
https://hgserver1.amc.nl/cgi-bin/r2/main.cgi

4
http://www.bioconductor.org/

5
http://autophagey.Iu/clusterring/index.html

6
http://www.gsea-msigdb.org/gsea/index.jsp

7
https://CRAN.R-project.org/package=factoextra

8
https://www.rdocumentation.org/packages/FactoMineR/versions/1.42

9
http://www.gsea-msigdb.org/gsea/index.jsp

10
http://biocc.hrbmu.edu.cn/TIP/index.jsp

## Supplementary Material

Supplementary Figure 1 The flowchart of the study.
