# Supplementary figures and images for: Identification of an Autophagy-Related Risk Signature Correlates With Immunophenotype and Predicts Immune Checkpoint Blockade Efficacy of Neuroblastoma
Source: Front Cell Dev Biol. 2021 Oct 21;9:731380. doi: 10.3389/fcell.2021.731380 (PMC8567030; doi:10.3389/fcell.2021.731380)

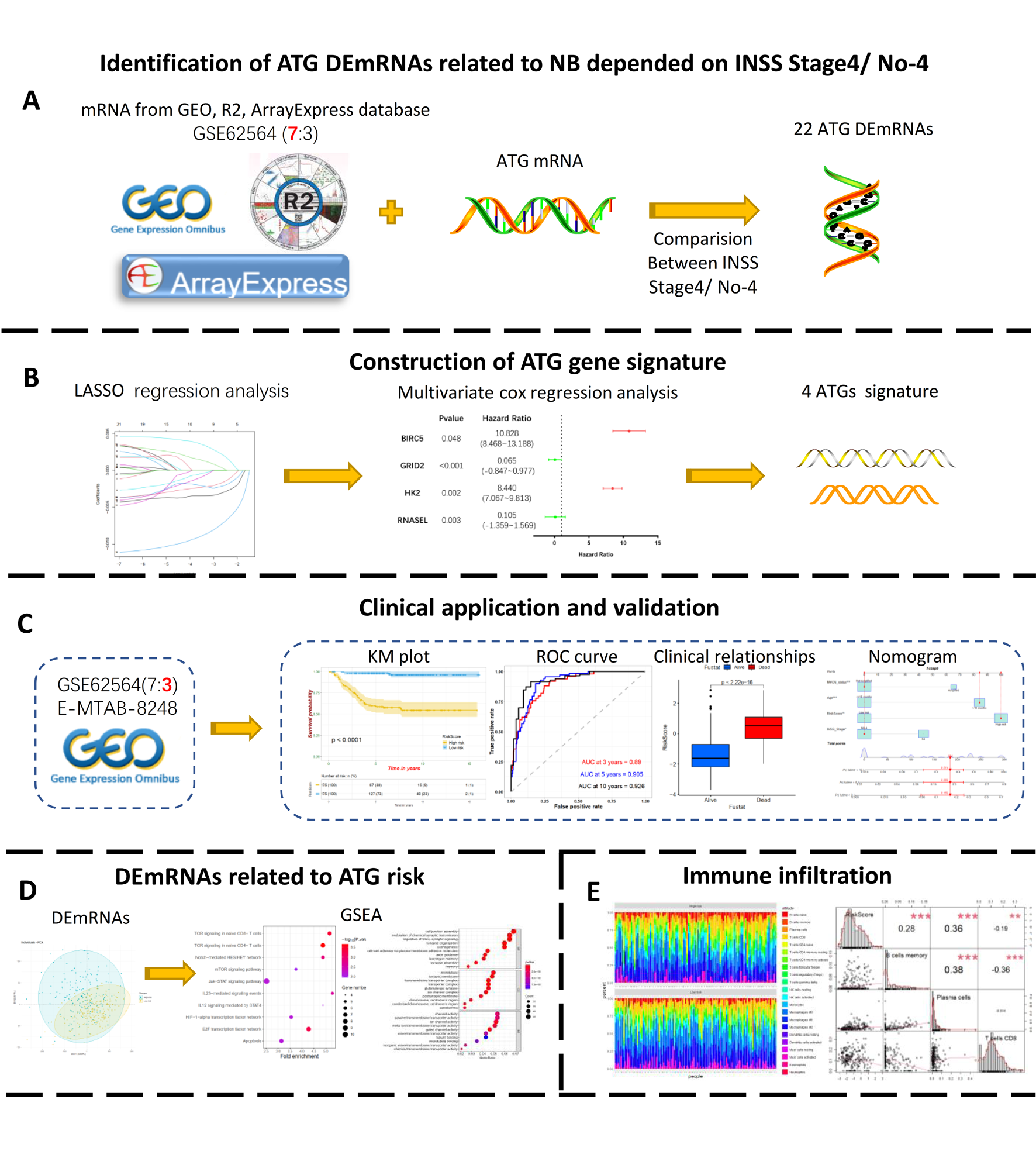

Supplement: Supplementary Figure 1 — The flowchart of the study. [file Image_1.TIF]
